# Effects of exercise combined with cervicothoracic spine self-mobilization on chronic non-specific neck pain

**DOI:** 10.1038/s41598-024-55181-8

**Published:** 2024-03-04

**Authors:** Ximei Sun, Liangwei Chai, Qiuyu Huang, Hua Zhou, Hua Liu

**Affiliations:** 1 Capital University of Physical Education and sports, Beijing, China; 2 West Yunnan University of Applied Sciences, Dali, Yunnan China; 3 Department of Orthopaedics, Peking University Third Hospital, Beijing, China

**Keywords:** Orthopaedics, Rehabilitation, Patient education, Musculoskeletal system

## Abstract

To investigate the short-term effects and differences between exercise alone and exercise combined with self-mobilization training on chronic non-specific neck pain (CNSNP). Thirty subjects who met the criteria were recruited and randomly assigned to the exercise training group, the exercise combined with cervical self-mobilization training group (ECCM), and the exercise combined with cervicothoracic self-mobilization training group (ECCTM). The exercise training group received 6 weeks of deep neck flexor under biofeedback and scapular stability training, and the other two groups received 6 weeks of cervical self-mobilization and cervicothoracic self-mobilization, respectively, in addition to exercise training. Neck pain, cervical range of motion (ROM), neck disability, strength and endurance of deep neck flexor and quality of life were assessed before and after 6 weeks of training. The study results showed that all the three training programs for 6 weeks increased the strength and endurance of deep neck flexor, increased cervical ROM, reduced pain, and improved neck function (P < 0.05). The exercise combined with self-mobilization two groups compared with only the exercise training group had better improvement in ROM of extension, lateral flexion, rotation and quality of life (P < 0.05). Compared with exercise alone and exercise combined with cervical self-mobilization training, the exercise combined with cervicothoracic self-mobilization training was the best in improving ROM of right lateral flexion (exercise training group vs ECCTM: P < 0.01, d = 1.61, ECCM vs ECCTM: P < 0.05, d = 1.14) and pain (exercise training group vs ECCTM: P < 0.05, d = 1.34, ECCM vs ECCTM: P < 0.05, d = 1.23). Deep flexor muscle and shoulder stability training can improve the endurance and strength of the deep flexor muscles of the neck and coordinate the movement patterns of the shoulder and neck. Self-mobilization techniques can promote improvements in cervical lateral flexion and rotation range of motion, alleviate neck disability and further improve quality of life. A combination of exercise and cervicothoracic self-mobilization training appears beneficial for the management of neck pain.

## Introduction

According to the Global Burden of Disease study, neck pain is the second most common musculoskeletal disorder and the third most common cause of a reduced life expectancy in men and women^1,2^. Although its symptoms may resolve, functional impairment remains, increasing the likelihood of recurrent acute neck pain that progresses to chronic neck pain. According to the literature, 54% of people experience neck pain, with 37% developing chronic neck pain^3^. Among people with chronic neck pain, those with unknown etiology and symptoms lasting more than 12 weeks are referred to as chronic non-specific neck pain (CNSNP)^4^. The prevalence of CNSNP is increasing, especially among younger people, due to the fast-paced nature of modern society and changes in living and working conditions^5^. Some studies had shown that the incidence of chronic neck pain in college students is proportional to the prolonged use of electronic devices and the various poor postures, which may be related to excessive stretching of the muscles and ligaments of the back neck, resulting in exerting excessive pressure on the intervertebral discs and surrounding tissues and leading to neck pain^6^. Most patients with neck pain will experience recurrence within 1–5 years, and one of the important reasons is the change in posture control^7^. The longus capitis and longus colli, deep neck flexors are considered important in postural adjustment^8^ and play a dynamic stabilizing role^9^. Studies using electromyography had shown that patients with neck pain experience delayed activation of deep and superficial neck muscles, which may be due to changes in neck movement control^10^. The impaired activation of the deep cervical flexor muscles and the subsequent potential for altered load distribution on the spine has been implicated in the pathogenesis and maintenance of recurrent painful cervical spine disorders^7^. An intervention or exercise program to improve deep cervical neck flexor function has been shown to reduce neck pain, enhance anterior cervical muscle endurance, and improve perceived cervical disability^11^.

In addition to therapeutic exercise, spinal mobilization is available for the management of nonspecific chronic neck pain. Spinal mobilizations of the specific dysfunctional segment or the remote segment, using passive distraction and gliding techniques passively to the joint surfaces in order to maintain or restore joint range of motion (ROM), could be used for the treatment of neck pain^12^. There is insufficient evidence that spinal manipulative therapy and cervical spine mobilization are superior to other standard treatments for patients with chronic neck pain^13^. Some studies have described a close and ergonomic that a relationship between the cervical and thoracic spine^14^. From the functional point of the entire spine, since the movement of the cervical spine includes the movement of the upper thoracic spine, hypomobility of the upper thoracic may be involved in inducing neck pain due to compensation^15^. Kim’s study showed that self mobilization in the upper thoracic showed significant improvement in terms of neck pain and ROM^16^. Some studies suggested that the multimodal treatment strategy was a good option for reducing disability in patients with CNSNP^12^. However, there was still a lack of relevant research on the combination of exercise and cervicothoracic spine self-mobilization for neck pain. The purpose of this study was to evaluate the short-term effects and differences between exercise alone and exercise combined with self-mobilization training on neck pain, ROM, strength and endurance of the deep cervical flexor, functional impairment, and quality of life.

## Methods

### Study design

This study used a single-blind, randomized, controlled design. We confirmed that informed consent to participate and informed consent to publish was obtained from all subjects and/or their legal guardians for publication of identifying information/images in an open-access online publication. The study fully adhered to the Declaration of Helsinki. This study was approved by Capital Sports University Ethics Committee (approval no. 2022A60). The clinical trial was registered at https://www.chictr.org.cn/bin/ser (21.07.2023) (registration number ChiCTR2300073801). All training programs were conducted at the Sports Rehabilitation Laboratory of the Capital University of Physical Education.

The subjects in this study were blinded to their group assignments. To ensure the integrity of the blinding, each subject was treated individually, and a blinding test was conducted at week 6 to assess its success. Furthermore, the involved researchers received training on the trial’s specifications and were required to adhere strictly to the principle of task separation to prevent the exchange of study information.

### Sample size

Some studies suggested that the pilot study results could be used as a basis for sample size calculation^17^. Kang et al.^18^ conducted a 4-week resistance training for the cervical spine and scapula of neck pain patients, using a pilot study to determine effect size and sample size. Similarly, Chen et al.^19^ calculated the sample size based on the mean and standard deviation of visual analogue score (VAS) pain scores for two groups after intervention in a pilot study. When analysis of variance was used, η^2^ could be used as the effect size to calculate the sample size^20^. Therefore, the result of pain improvement in the pilot study was used to calculate the sample size. In the pilot study, 15 subjects with CNSNP were recruited (5 subjects for each group) for a 6-week training program. Before intervention the VAS pain scores were as follows: the exercise training group for 5.4 ± 1.67; the exercise combined with cervical self-mobilization training group for 7.2 ± 1.64; the exercise combined with cervicothoracic self-mobilization training group for 6.2 ± 0.84. The pre-intervention data were normally distributed, and there were no significant differences among the three groups (P = 0.182). After the intervention the VAS pain scores were as follows: the exercise training group for 4.42 ± 1.08, the exercise combined with cervical self-mobilization training group for3.78 ± 1.26, and the exercise combined with cervicothoracic self-mobilization training group for 2.82 ± 0.70. The data were normally distributed, and there was a significant difference among the three groups after the intervention (P < 0.01). Conducting a one-way analysis of variance, an effect size of η^2^ was 0.67. In the formal experiment, a significance level of 0.05 and a power of 0.95 were set and calculated using G-power software (version 3.1.9.6; Franz Faul, University of Kiel, Kiel, Germany) software. So at least 21 subjects were required. Considering a 25% dropout rate, the sample size needed to be at least 27. Finally, 47 subjects were recruited in the study.

### Subjects

The 47 recruited subjects were included and excluded according to the following criteria^18^: (1) a history of chronic neck pain lasting at least 3 months localized to the back of the head and neck; (2)VAS pain score ≥ 3; (3) no treatment received within the past month; and (4) willingness to sign an informed consent form. Exclusion criteria were as follows: (1) a history of trauma or surgery to the head, neck, or other spinal segments; (2) severe cervical spondylotic radiculopathy, cervical spondylosis affecting the spinal cord, or cervical spondylosis affecting the vertebral artery; (3) presence of conditions such as osteoporosis, cervical tuberculosis, or tumors; and (4) cardiovascular or thoracic lung disease that could affect pulmonary ventilation and air exchange. Subjects were randomly assigned to one of three groups (n = 10 for each group) using the random number table method: the exercise training group, the exercise combined with cervical self-mobilization training group(ECCM), the exercise combined with cervicothoracic self-mobilization training group (ECCTM). Each group consisted of 10 subjects, and there were no significant differences in age and sex among the three groups.

### Interventions

Three groups received a training program for 6 weeks, three times a week, for 40 min each, for a total of 18 sessions. The exercise training only received 6 weeks of deep neck flexor under biofeedback and scapular stability training, and the other two groups received 6 weeks of cervical self-mobilization and cervicothoracic self-mobilization in addition to exercise training, respectively. The training program implementation was as follow. In the first session, the subjects were guided to perform deep neck flexor training under pressure biofeedback and scapular stability training. In additional, the two self- mobilization groups increased cervical and cervicothoracic training, respectively, and also received additional instruction and guidance on the use of mobilization belts until they fully mastered the training methods and could perform them independently. From the 2nd to the 9th session, the subjects performed the training program independently, only providing supervision and verbal feedback to ensure effective performance of the training. In the 10th session, the three training programs were gradually progressed under guidance until the subjects could perform the advanced training programs independently. From the 11th to the 18th session, the subjects performed all the training programs independently with only supervision and verbal feedback.

#### Exercise training group

**Figure 1:**
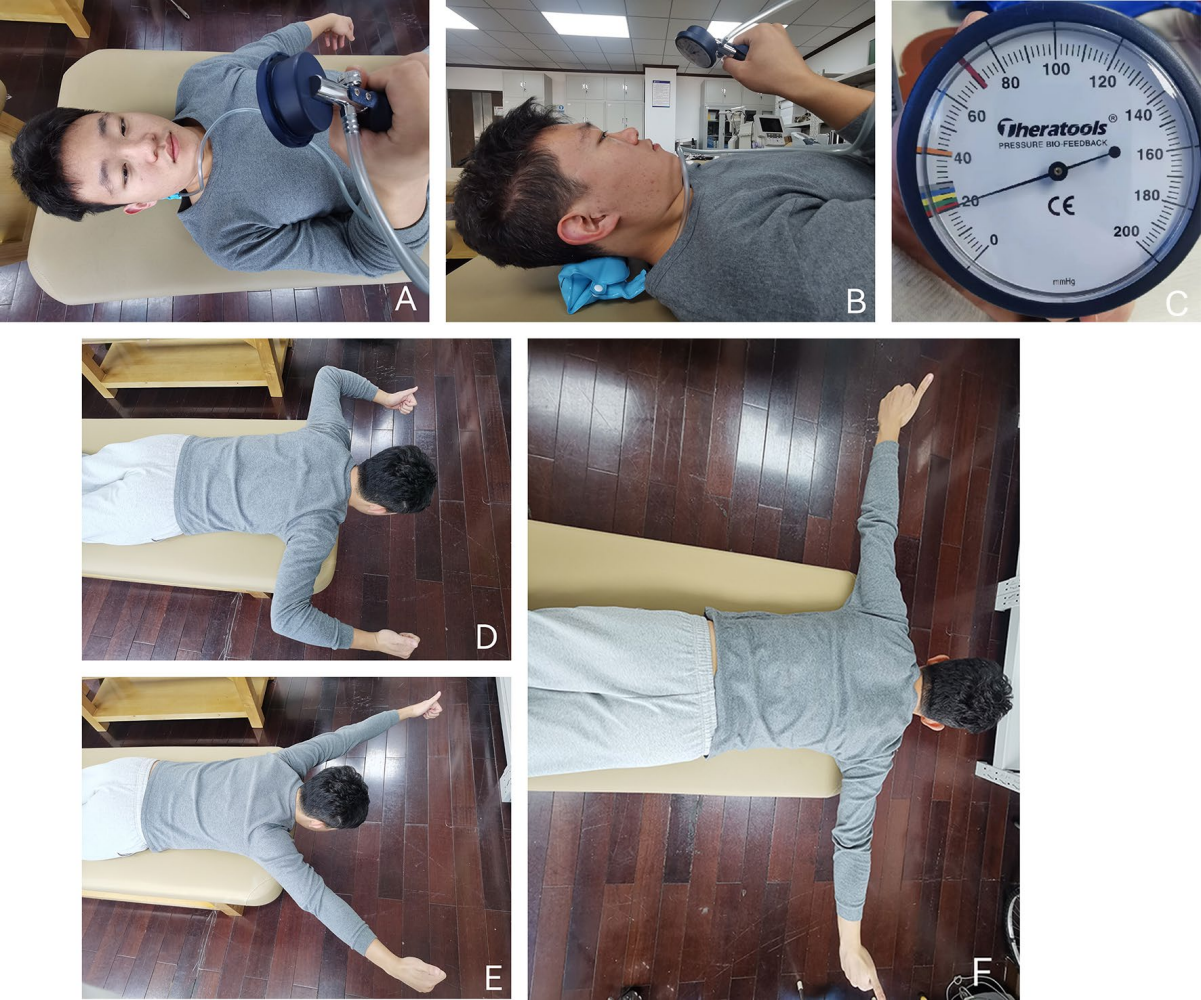
Exercise training group (**A**,**B**). Deep cervical flexor training. (**C**) Initial calibration of pressure biofeedback instrument. (**D**) Shoulder joint stability training “W” movement. (**E**) Shoulder joint stability training “Y” movement. (**F**) Shoulder joint stability training “T” movement.

The exercise training program mainly included the deep neck flexor exercise under pressure biofeedback and scapular stabilization. The exercise program for the deep neck flexor was as follows^5^: strength and endurance of deep neck flexor was exercised by a pressure biofeedback device (Stabilizer TM, Theatools Group, Inc.,Zhengzhou, CHN). The subject was placed in a supine position with the hips and knees flexed. Use the pressure biofeedback instrument from 20 mmHg for 20 s, rest for 10 s, and increase 2 mm Hg each time until 30 mmHg(Fig. 1A, B). The exercise training program was designed as three sets per week for the first three weeks and five sets per week for the last three weeks.

The exercise program for scapular stability was as follows^21^: the subject lay in the prone position with the head outside the bed and the arms extended to form the W, Y, and T shapes. For the W movement, the shoulder joint was abducted to 90°, the elbow joint was flexed to 90°, and the arms were slowly moved up and down. For the Y movement, the shoulder joint was elevated to 130°, the elbow joint were straightened,and the arms were swung up and down slowly. For the T movement, the shoulder joint was abducted to 90°, elbow joints straightened, and arms slowly moved up and down (Fig. 1D, E, F). In the first three weeks, do two sets of each exercise, 15 repetitions per set, three times a week. In the following three weeks, do each exercise 30 times per set.

#### Exercise combined with cervical self-mobilization training group

**Figure 2:**
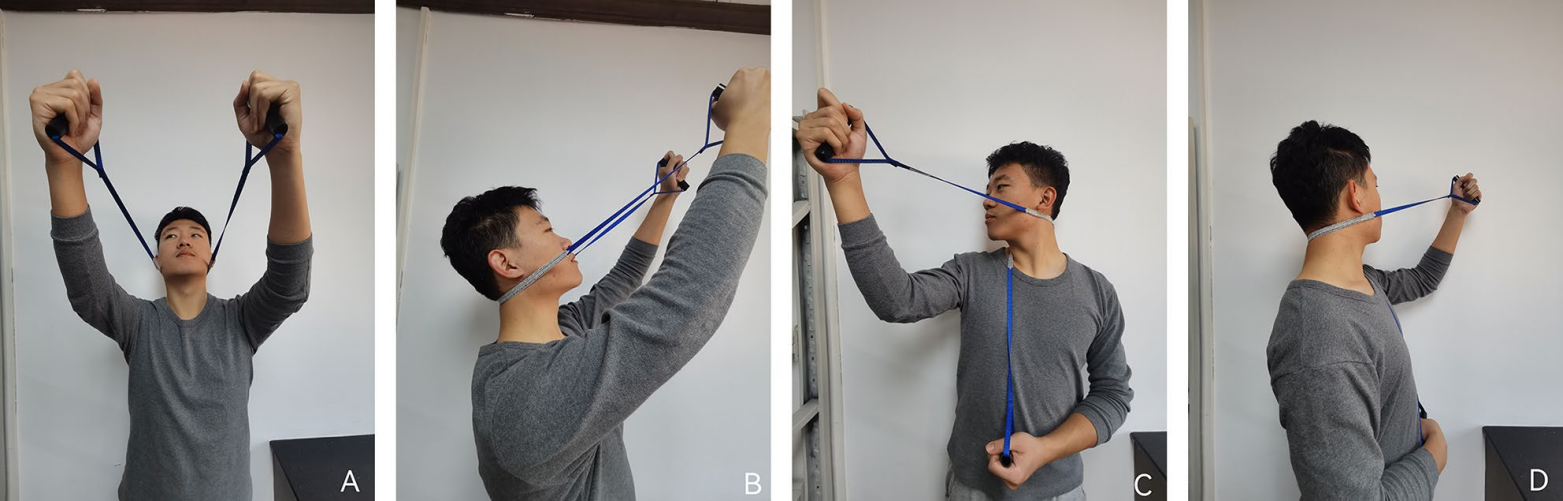
Exercise combined with cervical self-mobilization training group. (**A**,**B**) Cervical extension self-mobilization. (**C**) Cervical right rotation self-mobilization. (**D**) Cervical left rotation self-mobilization.

In this group, self-mobilization training for the cervical spine was combined with the same exercises as in the exercise training group. The program for dynamic cervical spine self-mobilization was as follows^22^: (1) cervical spine rotation: Using right rotation as an example, subjects were instructed to use a cervical self-joint mobilization band placed on the spinous processes of the affected cervical spine. Both hands held the opposite ends of the band and the left elbow was supported against the back of the chair to prevent rotation of the upper back. As the cervical spine rotated to the right, the band was held in the right hand and pulled in the direction of the facet joint plane of the cervical spine. At the end of the joint movement, moderate pressure was applied to the facet joint surface without causing pain; and (2) cervical extension: a folded band was placed on the spinous process of the affected cervical segment. Subjects pulled up the ends of the band with both hands, causing it to move along the treatment plane of the posterior cervical extension and facilitating increased vertebral sliding, thereby increasing mobility (Fig. 2A, B, C, D).

#### Exercise combined with cervicothoracic self-mobilization training group

**Figure 3:**
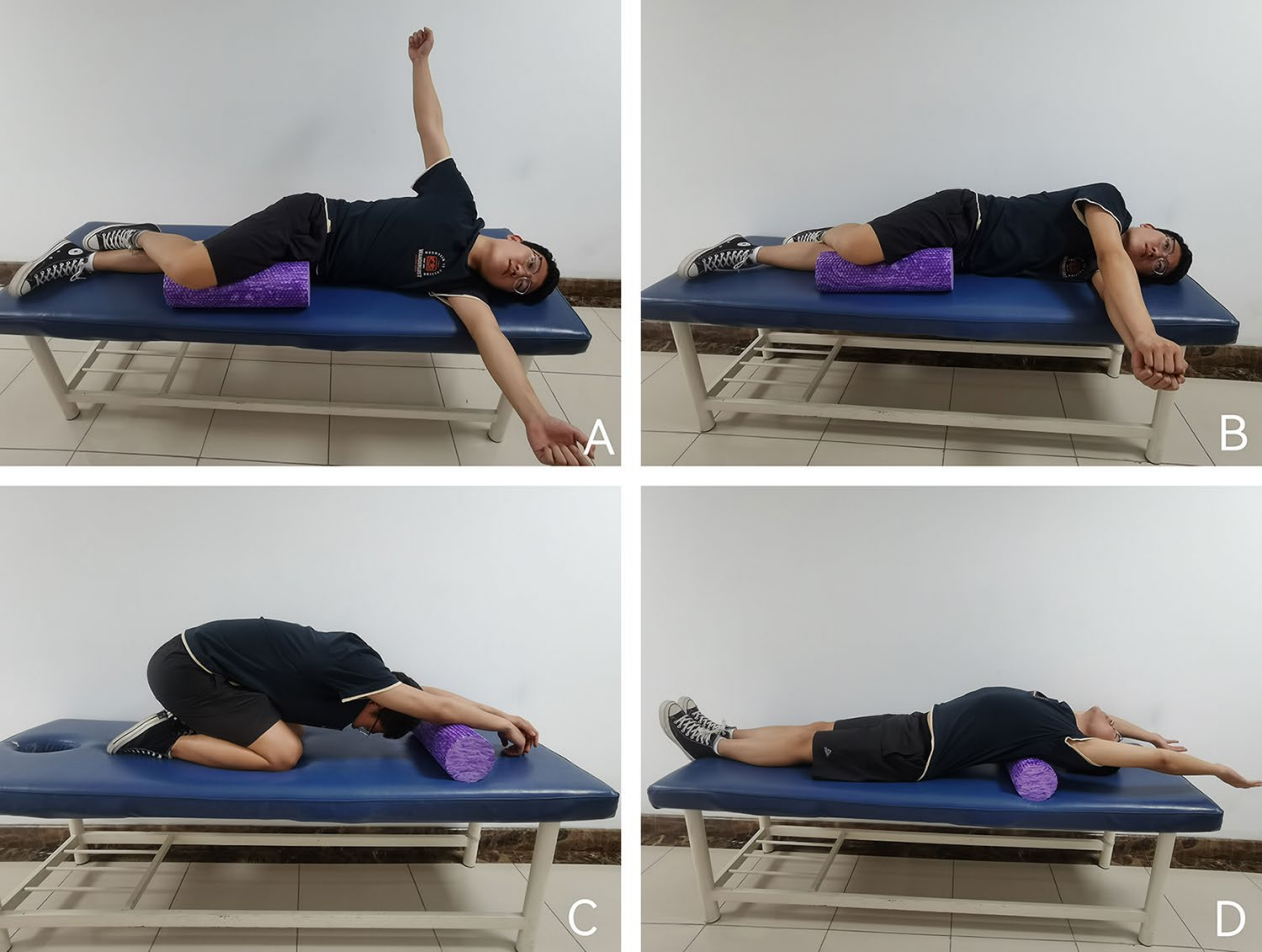
Exercise combined with cervicothoracic self-mobilization training group. (**A**,**B**) Thoracic rotation range of motion training. (**C**) Thoracic flexion range of motion training. (**D**) Thoracic extension range of motion training.

In this group, self-mobilization of the thoracic spine was added to the ECCM program. The thoracic spine mobilization exercises were as follows: (1) stretching of the thoracic spine using a foam roller. The subjects lay supine on a foam roller placed under the upper thoracic spine. They raised their arms, tilted their bodies backward, and directed their heads toward the ground. This position was maintained for 5–10 s, and the procedure was repeated for the next segment of the thoracic spine. This exercise was designed to improve thoracic spine mobility and relax the soft tissues in the thoracic region; (2) lateral rotation of the thoracic spine. The subjects lay with their hips and knees flexed at 90°. They placed the upper leg on a foam roller and the lower leg on a bed while maintaining stability of the pelvis and lumbar spine. The shoulder joint was flexed forward at 90°and the arms were held straight. The hands were joined to form the largest possible circle with the upper hand and the thoracic spine was rotated to its maximum ROM alternating between the left and right sides; and (3) prayer stretching with the foam roller. Subjects knelt and placed their buttocks on their heels. They placed their hands straight forward on the foam roller and kept their chest as close to the ground as possible during the movement. The goal was to sink the chest toward the ground while exhaling slowly. These self-mobilization exercises for the thoracic spine were designed to improve mobility, flexibility, and relaxation in the thoracic region (Fig. 3A, B, C, D).

### Outcome measures

Each of the three groups was evaluated by the same evaluator. Pain, six-way cervical ROM, cervical dysfunction, strength and endurance of deep cervical flexor, and quality of life were assessed before and after the 6-week intervention.

### VAS scores

Subjects were asked to indicate the intensity of their neck pain before and after the 6-week intervention on a 10-cm horizon scale, with 0 indicating no pain and 10 indicating excruciating pain. The reliability of the assessment tool (r = 0.97) was confirmed by Bijur et al. This assessment tool showed high intra-rater (r = 1.00) and inter-rater (r = 0.99) reliability^23^.

### Cervical ROM

Cervical ROM was assessed in six directional movements (flexion, extension, right and left lateral flexion, right and left rotation) using a cervical goniometer (CROM Deluxe, Thea-tools Group, Inc., Zhengzhou, CHN), which had a high degree of repeatability with an inter-rater reliability of 0.89–0.98^24^.

### Neck disability index

The NDI measures disability in the neck. The NDI is a self-report instrument for assessing disability in subjects with neck pain (cervical spine dysfunction index = total score of each item/number of items completed by the subject × 5 × 100%)^25^. The intraclass correlation coefficient (r = 0.92) and the evaluation method of criterion reliability by internal consistency and Cronbach’s alpha coefficient (a = 0.96) demonstrated a high degree of reliability^23^.

### Strength and endurance of deep neck flexor

In clinical practice, pressure biofeedback devices have primarily been used to test and train the deep neck flexor muscles to improve endurance^26^. Subjects were placed in the supine position, and the balloon of the pressure biofeedback instrument was placed under the occipital bone and inflated to 20 mm Hg. The subjects performed craniocervical flexion, pressed the pillow against the ball and applied pressure. When the pressure reached 22 mm Hg, the subject held the posture for 10 s, and then rested for 10 s. The procedure was repeated at the pressures of 24, 26, 28, and 30 mm Hg. If 10 s was not maintained at any stage, the test was terminated and the pressure value was recorded^27^. This test has a high degree of repeatability, with intra- and inter-observer reliability ranging from 0.81 to 0.93^15^.

The strength of deep neck flexor was evaluated by measuring the maximum voluntary isometric contraction using dynamometers (Micro FET3, West Jordan, Ut 84088). This dynamo-meters measurement had good test–retest reliability (intraclass correlation coefficient, 0.70–0.94)^28^. This dynamometer was conducted under the seat During the test, the evaluator placed the dynamo-meter behind the chin, provided resistance, and instructed the subject to make an effort to perform neck flexion isometric contractions while keeping the thoracic spine in a neutral position to avoid compensation. Measurements were taken twice and the average value was taken.

### Quality of life

The Short Form-36 Health Survey was used to assess quality of life^29^. The physical component scores(PCS, physical functioning, role limitations due to physical health, bodily pain, general health perceptions) and mental component scores(MCS, vitality, social functioning, role limitations due to emotional problems, mental health) were calculated separately^30^. It had shown significant correlations (r = − 0.74, − 0.67) between the physical and mental component scores^31^.The calculation process for PCS and MCS had been previously described by Taft et al.^30,32^.

### Statistical analysis

The data were analyzed using SPSS 22.0 (version 22.0; SPSS Inc., Armonk, NY, USA). The data were presented as mean ± standard deviation (M ± SD). Normality tests and homogeneity of variance tests were performed first. If normal, one-way analysis of variance was used for baseline comparison; otherwise, the rank sum test was used. A two-way repeated-measures analysis of variance (ANOVA) test was performed to evaluate the main effects of time (before and after intervention), group and the interaction effect between time with group. Note that for the inter-subject variables comparison, one-way ANOVA with Tukey’s post-hoc test was used as the test for between-group variables for the comparison of between-subject variables, whereas a paired t-test was used for the comparison of within-subject variables.

Cohen’s d was used to calculate the magnitude of the effect size between two means. An effect size of d from 0.1 to 0.29 was considered a small effect size; d from 0.3 to 0.49 was considered a moderate effect size; d from 0.5 to 0.69 was considered a large effect size, and d ≥ 0.7 was considered a very large effect size^33^. The 95% confidence interval (CI) around the effect size was also calculated to assess the precision of the effect size precision^34^. The significance level was set at P < 0.05.

## Results

**Figure 4:**
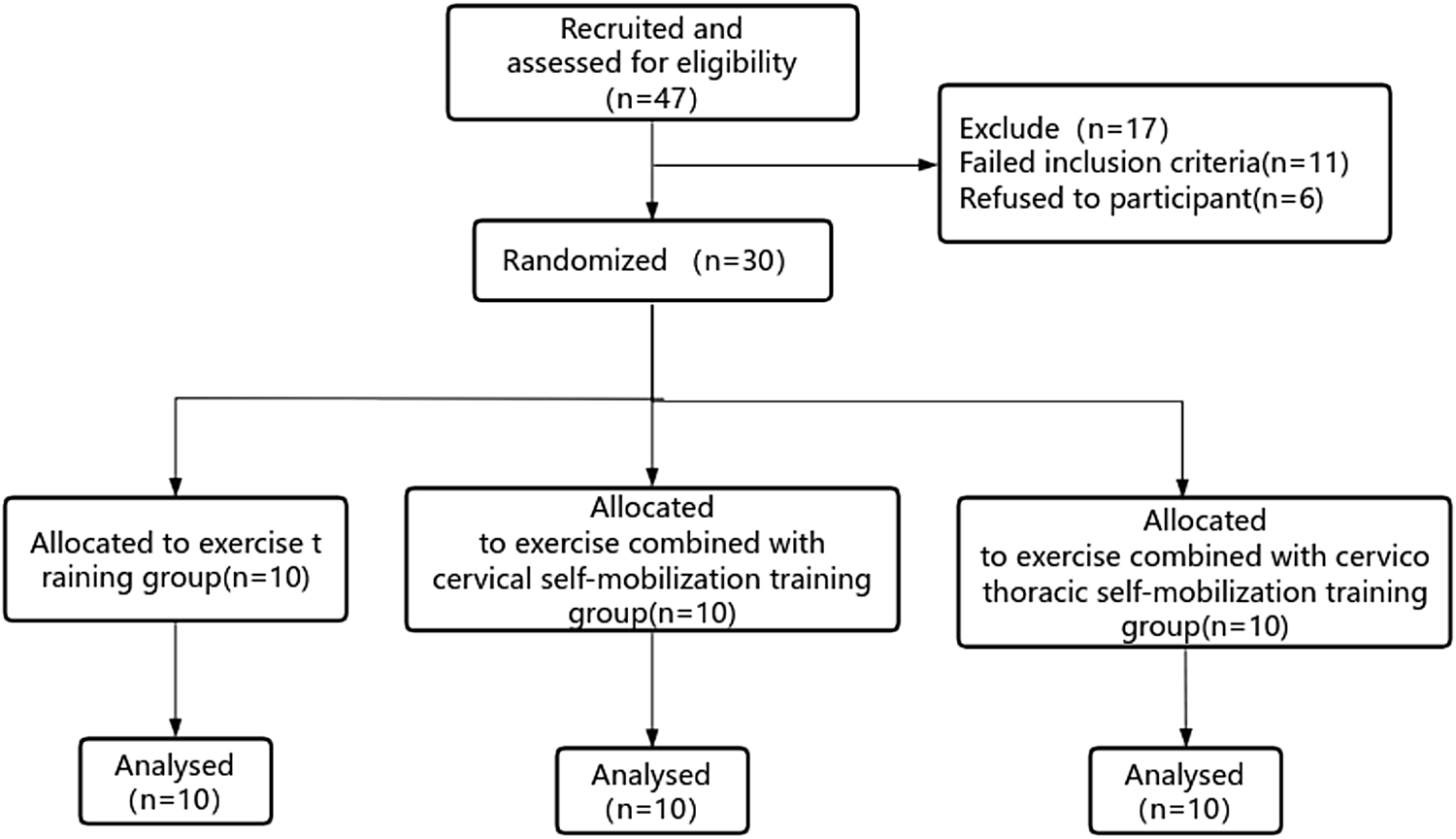
Article inclusion flow chart.

Forty-seven subjects were screened for eligibility. 17 were excluded, of which 11 did not meet the inclusion criteria and 6 refused to participate due to personal reasons. 30 subjects (10 in each group) completed all training programs and were included in the final analysis (Fig. 4). Each outcome measure included in the analysis was normally distributed and there was no significant difference between the three groups before intervention (Table 1).

**Table 1:** Overall outcomes of the three groups before and after 6-week intervention.

Variables	Group	Before invention	After invention	t	*P*
VAS	Exercise training group	5.70 ± 2.06	3.30 ± 0.67	4.811	< 0.001
	ECCM	5.70 ± 1.70	3.20 ± 0.63	5.622	< 0.001
	ECCTM	5.80 ± 1.40	2.30 ± 0.82	8.720	< 0.001
Flexion ROM	Exercise training group	26.52 ± 6.90	37.20 ± 6.22	− 4.930	0.001
	ECCM	26.76 ± 6.72	37.40 ± 5.68	− 4.598	0.001
	ECCTM	28.70 ± 7.04	40.34 ± 3.30	− 7.684	0.000
Extension ROM	Exercise training group	39.56 ± 7.50	44.02 ± 6.89	− 2.869	0.018
	ECCM	42.40 ± 5.80	52.07 ± 3.64	− 4.583	0.001
	ECCTM	42.40 ± 5.79	51.59 ± 4.87	− 4.364	0.002
Left lateral flexion ROM	Exercise training group	36.27 ± 8.14	41.33 ± 4.67	− 1.792	0.107
	ECCM	34.37 ± 6.42	43.51 ± 3.53	− 4.166	0.002
	ECCTM	37.92 ± 5.45	45.76 ± 2.47	− 4.152	0.002
Right lateral flexion ROM	Exercise training group	34.84 ± 8.29	38.57 ± 4.46	− 1.839	0.099
	ECCM	34.68 ± 7.75	41.31 ± 3.39	− 3.517	0.007
	ECCTM	38.07 ± 6.11	46.11 ± 4.89	− 4.652	0.001
Left rotation ROM	Exercise training group	46.32 ± 8.82	47.25 ± 6.31	− 0.362	0.726
	ECCM	42.63 ± 7.67	49.41 ± 4.34	− 4.019	0.003
	ECCTM	42.75 ± 7.57	54.72 ± 5.61	− 4.628	0.001
Right rotation ROM	Exercise training group	50.58 ± 4.40	50.08 ± 2.51	0.371	0.719
	ECCM	49.03 ± 6.42	53.80 ± 3.80	− 3.387	0.008
	ECCTM	49.23 ± 6.75	58.02 ± 6.27	− 3.479	0.007
DCF endurance (mmHg)	Exercise training group	24.20 ± 2.74	35.40 ± 4.22	− 8.806	< 0.001
	ECCM	24.60 ± 2.67	35.20 ± 3.79	− 9.165	< 0.001
	ECCTM	23.20 ± 1.40	36.20 ± 8.72	− 4.867	< 0.001
DCF strength (N)	Exercise training group	54.70 ± 10.51	73.50 ± 16.84	− 5.681	0.008
	ECCM	54.20 ± 12.88	73.30 ± 19.62	2.343	0.019
	ECCTM	56.30 ± 17.23	78.50 ± 15.63	− 16.884	0.007
Neck disability index	Exercise training group	0.53 ± 0.14	0.42 ± 0.07	2.127	0.048
	ECCM	0.44 ± 0.08	0.39 ± 0.05	2.343	0.044
	ECCTM	0.45 ± 0.10	0.36 ± 0.07	2.234	0.038
Quality of life (PCS)	Exercise training group	0.11 ± 0.10	0.17 ± 0.10	− 1.287	0.115
	ECCM	0.12 ± 0.08	0.23 ± 0.07	− 3.100	0.006
	ECCTM	0.11 ± 0.08	0.26 ± 0.06	− 4.782	< 0.001
Quality of life (MCS)	Exercise training group	− 0.34 ± 0.03	− 0.31 ± 0.04	− 1.842	0.099
	ECCM	− 0.31 ± 0.04	− 0.20 ± 0.16	− 3.187	0.011
	ECCTM	− 0.31 ± 0.06	− 0.19 ± 0.16	− 3.609	0.006

*ROM* range of motion, *ECCM* exercise combined with cervical self-mobilization training group, *ECCTM* exercise combined with cervicothoracic self-mobilization training group, *DCF* deep cervical flexor, *PCS* physical component summary, *MCS* mental component summary.

### VAS scores

After 6 weeks of intervention there was no significant interaction effect between group and time for the VAS score (F = 0.973, P = 0.388). The ANOVA results showed indicated a significant simple main effect for the group (F = 6.294, P = 0.006), while the mean VAS score of the ECCTM group was significantly lower than that of the other two groups (ECCTM vs Exercise training: P = 0.014, d = 1.34, ECCTM vs ECCM: P = 0.014, d = 1.34) (Tables 1, 2).

**Table 2:** Intergroup comparasions of outcomes among the three groups after 6-week intervention.

Variables	Group	MD (95% CI)	*P*	d
VAS	Exercise training group vs ECCM	0.00 (− 0.83 to 0.83)	1.000	0.15
	Exercise training group vs ECCTM	1.00 (0.17 to 1.83)	0.014	1.34
	ECCM vs ECCTM	1.00 (0.17 to 1.83)	0.014	1.23
Flexion ROM	Exercise training group vs ECCM	− 0.20 (− 6.32 to 5.92)	1.000	0.03
	Exercise training group vs ECCTM	− 3.14 (− 9.26 to 2.98 )	0.605	0.63
	ECCM vs ECCTM	− 2.94 (− 9.06 to 3.18)	0.693	0.63
Extension ROM	Exercise training group vs ECCM	− 8.05 (− 12.91 to − 3.18)	0.006	1.46
	Exercise training group vs ECCTM	− 7.56 (− 12.43 to − 2.70)	0.011	1.27
	ECCM vs ECCTM	− 0.48 (− 4.38 to 5.34)	1.000	0.11
Left lateral flexion ROM	Exercise training group vs ECCM	− 2.18 (− 6.36 to 2.01)	0.588	0.53
	Exercise training group vs ECCTM	− 4.42 (− 8.79 to − 1.06)	0.036	1.19
	ECCM vs ECCTM	− 2.25 (− 5.61 to 1.12)	0.546	0.74
Right lateral flexion ROM	Exercise training group vs ECCM	− 2.73 (− 6.29 to 0.81)	0.375	0.69
	Exercise training group vs ECCTM	− 7.53 (11.08 to − 3.99)	0.001	1.61
	ECCM vs ECCTM	− 4.80 (− 8.34 to − 1.25)	0.030	1.14
Left rotation ROM	Exercise training group vs ECCM	− 2.16 (− 7.18 to 2.87)	1.000	0.40
	Exercise training group vs ECCTM	− 7.47 (12.50 to − 2.44)	0.015	1.25
	ECCM vs ECCTM	− 5.31 (− 10.34 to − 0.28)	0.118	1.06
Right rotation ROM	Exercise training group vs ECCM	− 3.71 (− 7.81 to 0.40)	0.224	1.16
	Exercise training group vs ECCTM	− 7.94 (− 12.04 to − 3.83)	0.001	1.66
	ECCM vs ECCTM	− 4.23 (− 8.33 to − 0.13)	0.131	0.81
DCF endurance (mmHg)	Exercise training group vs ECCM	0.20 (− 5.31 to 5.71)	1.000	0.05
	Exercise training group vs ECCTM	− 0.80 (− 6.31 to 4.71)	1.000	0.12
	ECCM vs ECCTM	− 1.00 (− 6.51 to 4.51)	1.000	0.15
DCF strength (N)	Exercise training group vs ECCM	0.20 (− 15.81 to 16.21)	1.000	0.01
	Exercise training group vs ECCTM	− 5.0 (− 21.01 to 11.01)	1.000	0.31
	ECCM vs ECCTM	− 5.20 (− 21.21 to 10.81)	1.000	0.29
Neck disability index	Exercise training group vs ECCM	0.03 (− 0.03 to 0.09)	0.922	0.49
	Exercise training group vs ECCTM	0.06 (0.00 to 0.12)	0.122	0.86
	ECCM vs ECCTM	0.03 (− 0.02 to 0.09)	0.831	0.49
Quality of life (PCS)	Exercise training group vs ECCM	− 0.05 (− 0.13 to − 0.02)	0.361	0.70
	Exercise training group vs ECCTM	− 0.09 (− 0.16 to − 0.02)	0.037	1.09
	ECCM vs ECCTM	− 0.04 (− 0.11 to − 0.03)	0.870	0.46
Quality of life (MCS)	Exercise training group vs ECCM	− 0.11 (− 0.23 to 0.01)	0.236	0.94
	Exercise training group vs ECCTM	− 0.11 (− 0.24 to 0.01)	0.207	1.03
	ECCM vs ECCTM	− 0.00 (− 0.13 to 0.12)	1.000	0.06

*ROM* range of motion, *ECCM* exercise combined with cervical self-mobilization training group, *ECCTM* exercise combined with cervicothoracic self-mobilization training group, *DCF* deep cervical flexor, *PCS* physical component summary, *MCS* mental component summary.

### Cervical ROM

There was no significant interaction effect between group and time in the cervical flexion (F = 0.034, P = 0.967), extension (F = 2.266, P = 0.134), and left (F = 3.118, P = 0.070) and right (F = 3.183, P = 0.067) lateral flexion ROM; however, there was a significant interaction effect between group and time in the left (F = 8.762, P = 0.002) and right (F = 4.702, P = 0.024) rotation ROM. The ANOVA results showed indicated a significant simple main effect of group in extension (F = 7.239, P = 0.003), left (F = 3.635, P = 0.040) and right (F = 9.731, P = 0.001) lateral flexion motion, left rotation (F = 4.916, P = 0.015), and right rotation (F = 7.886, P = 0.002). The comparisons between groups showed that the ECCTM group had medium to large effect sizes compared to the other two groups (flexion: ECCTM vs exercise training group, P = 0.605, d = 0.63; ECCTM vs ECCM, P = 0.693, d = 0.63; left lateral flexion: ECCTM vs exercise training group: P = 0.036, d = 1.19, ECCTM vs ECCM: P = 0.546, d = 0.74; right lateral flexion: ECCTM vs exercise training group: P = 0.001, d = 1.61, ECCTM vs ECCM: P = 0.030, d = 1.14; left rotation: ECCTM vs exercise training group: P = 0.015, d = 1.25, ECCTM vs ECCM:: P = 0.118, d = 1.06; right rotation: ECCTM vs exercise training group: P = 0.001, d = 1.66, ECCTM vs ECCM: P = 0.131, d = 0.81). In contrast, there was no significant difference in extension ROM between ECCTM and ECCM group (P = 1.000, d = 0.11) (Tables 1, 2).

### Strength and endurance of deep neck flexor

After 6 weeks of intervention there was no significant interaction effect for the strength and endurance of deep neck flexor between group and time(endurance: F = 0.452, P = 0.520; strength: F = 0.115, P = 0.762). And the strength (P < 0.05) and endurance (P < 0.01) of the deep neck flexor significantly increased after 6 weeks of intervention compared with before intervention (Tables 1, 2).

### Neck disability index

After 6 weeks of intervention there was no significant interaction effect between groups and time for the neck disability index (F = 0.460, P = 0.521). The neck disability index improved in the ECCTM and the exercise training group after 6 weeks intervention compared with before the intervention(P < 0.05), and the improvement of the ECCTM group was better than that in the exercise training group (P = 0.122, d = 0.86) (Tables 1, 2).

### Quality of life

After 6 weeks of intervention there was no significant interaction effect between group and time for quality of life (PCS: F = 3.648, P = 0.040; MCS: F = 2.310, P = 0.119). The ANOVA showed a significant simple main effect of group on PCS (F = 3.648, P = 0.040). In addition, PCS increased significantly in the ECCTM compared with the exercise training group (P = 0.037, d = 1.09) (Tables 1, 2).

## Discussion

The incidence rate of chronic non-specific neck pain is increasing^5^. Many young people are affected by frequent and long term use of electronic products, a sedentary lifestyle and prolonged postures which may be related to the onset and recurrence of neck pain. Therefore, a multimodal self-management program is very important for CNSNP. The study results showed that all three exercise programs increased deep neck flexor strength and endurance and ROM, reduce pain, and improved neck disability. The exercise combined with self-mobilization two groups showed significant advantages in improving ROM of extension, lateral flexion, rotation and quality of life, and the exercise combined with cervicothoracic self-mobilization group was the best in improving ROM of right lateral flexion and relieving pain among the three groups.

### The effect of the three training programs on ROM

Patients with neck pain are often associated with limited ROM, especially with ROM of extension and rotation^35^. In this study, all the three training programs used the exercises including the deep neck flexor exercise under pressure biofeedback and active movement of the shoulder joint complex to keep the head off the bed, and then effectively improved flexion and extension mobility. Consistent with the systematic review by Blomgren et al.^21^, the deep neck flexor exercise increased the active joint mobility of neck pain patients. This study also showed that addition of cervical and upper thoracic spine self-mobilization training in addition to exercise could better improve ROM of rotation and lateral flexion. Lopez-Lopez^36^ demonstrated that applying joint mobilization therapy four times for 2 weeks increased the flexion, extension, and rotation ROM of the neck by 8.3°, 13.3°, and 12.6°, respectively. Aquin’s study also showed that four weeks of mobilization therapy improved the six directions of cervical spine joint mobility (flexion, extension, rotation, and lateral flexion) in patients with neck pain^20^. The self-mobilization technique of the cervical spine involves small, localized sliding movements of the facet joints^37^, which can stimulate stimulate sensory neurons around the joints, and improve neuromuscular coordination. Applying appropriate tension and pressure through the self-mobiization band to stretch and relax the muscles around the neck, reducing muscle tension and stiffness, further improved joint mobility^38^.

The self-mobilization technique acts on the cervical spine segments with pain disorders by applying pressure to the joint plane during movement and at the end of joint activity to promote slight sliding increases to increase the joint mobility of the cervical spine^39^. By adjusting the space between the joints of cervical spine, reducing the pressure between the joints, stretching tight muscles, and adjusting the tension and coordination of the muscles around the neck, this technique increases the mobility of the cervical spine joints^40^. Our study also found similar results, and showed that adding self-mobilization into exercise could improve 4.77°–11.97°rotation ROM and 6.63°–9.14°lateral flexion ROM more effectively than exercise alone, and the cervicothoracic self-mobilization group showed better improvement in right lateral flexion ROM, which may be related to handedness. Patients affected with neck pain who frequently use their right hand had a lower surface electromyography frequency in the right upper trapezius, which was often in a contracted and tense state^41^. Through self-mobilization training of the cervicothoracic spine, muscle stretching and reducing stiffness reduction may increase joint mobility in right lateral flexion after intervention.

### The effect of the three training programs on pain

The deep flexor muscles of the neck are important for supporting the head and neck, maintaining neck stability, and controlling the acceleration of head movement. Tolentino et al. had shown that the strength of the neck deep flexor muscles was negatively correlated with neck pain and associated with functional impairment^42^. Decreased strength and endurance of the neck deep flexor muscles, which may increase the risk of neck injury and pain. Iqbal et al. demonstrated that a 6-week training program targeting the neck deep flexor muscles significantly reduced neck pain^43^. Patients with chronic neck and shoulder pain often have decreased scapular retraction, upward scapula rotation, and increased posterior tilt, resulting in altered scapular motion^44^. Abnormal shoulder motion patterns associated with abnormal stress and pressure distribution may affect the posture and movement patterns of the neck and upper back, which can lead to excessive tension and wear on the neck joints and soft tissues, resulting in neck pain^45^. Consistent with Hwang's study, a week self-mobilization of the upper spine could effectively reduce pain scores^16^, possibly by reducing TNF-α and IL-1β production through self-mobilization, down regulating chemokines, and inducing cortisol release to alleviate pain^46^. In addition, mobilization can increase the pain threshold by 10% ^38^, excite sympathetic nerves, and inhibit endogenous pain^47^. This study utilized self-mobilization techniques for the cervical spine were used to correct abnormal biomechanics, reduce pressure and compensation on the cervical spine, and further improve nerve conduction by applying pressure to the joint ends, increasing control, and reducing pain.

Some studies had found a significant association between reduced upper thoracic spine mobility and neck pain. Our study results showed that self-mobilization of the neck and thoracic spine combined with exercise was more effective in improving neck pain. Consistent with the previous study, a systematic review mentioned that mobilization targeting the thoracic spine in patients with mechanical neck pain patients over a 3-week period had a significant effect on pain reduction (0.38–4.03)^48^. Due to the similarity in morphology between the upper thoracic spine and the cervical spine, as well as the consistent direction of movement coupling, self-mobilization of the upper thoracic spine can increase its flexibility, allow movement of the cervical spine to the end ROM, and then promote restoration of normal biomechanics in this area. It could also reduce the mechanical stress distributed on the cervical spine, relieve the load on the cervical spine, and thereby reduce pain^49^.

### The effects of the three training programs on the strength and endurance of the deep neck flexor, neck disability, and quality of life

The deep flexor muscles of the neck in patients with neck pain are often in an inhibited state, affecting the length and tension relationship of the related neck tissues^20^. A 6-week training program focused on neck deep flexor muscle strength and endurance could increase recruitment of the deep flexor muscles, and reduce excessive activation of the sternocleidomastoid and anterior scalene muscles^50^. In this study, all the three training interventions included 6 weeks of pressure biofeedback training for the neck deep flexor muscles,which resulted in a significant improvements in neck deep flexor muscle strength (18.8–22.2 N) and endurance (10.6–13.0 mm Hg). These results were consistent with the findings of a systematic review, which indicated that neck deep flexor muscle training, compared to proprioceptive training, muscle stretching, and a non-intervention control group, could effectively improve strength and endurance, enhances head and neck posture, and improves neuromuscular coordination^21^. In addition, Iqbal et al. also found that a 6-week pressure biofeedback training for the neck deep flexor muscles significantly improved neck deep flexor muscle endurance, neck pain scores, and neck functional disability in patients with chronic neck pain^43^. In addition, this study also used "W", "Y", "T" exercises to train the muscles around the scapula, which can inhibit excessive activation of the upper trapezius muscle, facilitate normal movement patterns, realign the cervical spine^51^, and further improve the quality of life.

### Study limitations

This study only examined the neck muscle strength, cervical ROM, pain, and the effects of different training programs in college students with neck pain, without collecting information on sitting posture information. We will continue to focus on the neck posture and sitting posture changes in special groups such as adolescents and college students, and the neck pain they may cause in future research. This study did not assess muscle activity, and future studies should add electromyography to muscle measurements. Long-term effects were not explored in this study, however chronic non-specific neck pain is chronic and prone to recurrence, and completion of the home-based training program completion and neck pain recurrence rates should be evaluated in future studies.

## Conclusions

Deep flexor muscle and shoulder stability training can improve the endurance and strength of the deep flexor muscles of the neck and coordinate the movement patterns of the shoulder and neck. Self-mobilization techniques can promote improvements in cervical lateral flexion and rotation range of motion, alleviate neck disability and further improve quality of life. A combination of exercise and cervicothoracic self-mobilization training appears beneficial for the management of neck pain.

## Data Availability

The data sets generated and analyzed during the study are available from the corresponding author upon reasonable request.
